# How sustainable are self-help interventions in the post-funding period? Qualitative findings from integrated leprosy communities in Nigeria

**DOI:** 10.1093/inthealth/ihaf140

**Published:** 2025-12-19

**Authors:** Onaedo Ilozumba, Paul Tsaku, Sopna Choudhury, Sunday Udo, Pius Sunday, Antje Lindenmeyer, Richard Lilford, Frances Griffiths

**Affiliations:** Institute of Applied Health Research, College of Medical and Dental Sciences, University of Birmingham, Edgbaston, Birmingham B15 2TT, UK; The Leprosy Mission Nigeria, 9 Kings Drive, Fort Royal Estate, Abuja, Nigeria; Institute of Applied Health Research, College of Medical and Dental Sciences, University of Birmingham, Edgbaston, Birmingham B15 2TT, UK; The Leprosy Mission Nigeria, 9 Kings Drive, Fort Royal Estate, Abuja, Nigeria; The Leprosy Mission Nigeria, 9 Kings Drive, Fort Royal Estate, Abuja, Nigeria; Institute of Applied Health Research, College of Medical and Dental Sciences, University of Birmingham, Edgbaston, Birmingham B15 2TT, UK; Institute of Applied Health Research, College of Medical and Dental Sciences, University of Birmingham, Edgbaston, Birmingham B15 2TT, UK; Division of Health Sciences, Warwick Medical School, University of Warwick, Coventry, UK

**Keywords:** leprosy, low- and middle-income countries, neglected tropical diseases, self-help groups, sustainability

## Abstract

**Background:**

Time-limited, externally funded self-help groups (receiving training, equipment and access to seeds) are typically evaluated during the funding period. However, their sustainability beyond this phase remains understudied.

**Methods:**

This retrospective qualitative study explored the post-funding sustainability of two 6-y self-help interventions (2013–2019) for groups in integrated leprosy communities in central Nigeria. Semi-structured interviews were conducted with 40 stakeholders (beneficiaries, implementers and funders) and supplemented by document analysis.

**Results:**

Findings revealed that most activities ceased after funding ended, with limited evidence of sustained benefits. In one community, sewing initiatives persisted, while the other showed no lasting impact. Challenges included inadequate maintenance of provided equipment (e.g. sewing machines, tricycles) and systemic barriers such as insecure land tenure and limited educational access.

**Conclusions:**

The study highlights the fragility of externally funded self-help models in marginalised communities. Sustainability requires integration with broader economic systems and infrastructure support. Funders should prioritise long-term planning alongside short-term interventions.

## Introduction

There is extensive literature on the development economics of local interventions to improve people’s economic circumstances.^1–3^ Likewise, there is a large body of literature dating back two centuries on the link between poverty and poor health.^4–6^ There is also literature on the link between economic interventions and health.^7–9^ These strands of thought are brought together in compound interventions that combine interventions to promote health with interventions to promote economic development. Such interventions are often referred to as ‘self-help’, thereby distinguishing them from purely health-related community interventions, referred to as ‘self-care’.^10,11^ Self-help interventions have often been used in leprosy, where self-care of people with (or at risk of) leprosy ulcers is combined with measures for economic empowerment.^12,13^ Since the prevalence of leprosy has declined over the last half century, these groups have often expanded to include a wider range of people with disabilities or simply people who are ‘marginalised’ through extreme poverty.^13^

In our protocol, we provide a comprehensive definition of self-help groups (SHGs) and sustainability.^13^ By sustainability, we mean the continuation of core income-generating or livelihood activities, and their associated benefits, that were initiated under external support, beyond the end of that support. While there is extensive literature on the general sustainability of development programs, including microfinance and community health initiatives, longitudinal studies that track the post-funding sustainability of SHGs, particularly in the context of leprosy and neglected tropical diseases, are rare. A review we conducted found only one such study focusing on SHGs in a related context.^14^ Our study aims to address this specific gap.

Our study is based on two self-help programs in Nigeria, described below. In contrast to most SHGs, which target a group of disabled and/or marginalised people in a broader community, such as a village, the self-help interventions described here were implemented among two communities originally established to cater to people affected by leprosy. Thus our study of sustainability focuses on a specific time-limited self-help intervention superimposed on communities that had already been established to improve the well-being of people affected by leprosy (and their descendants).

## Methods

### Study design

This was a qualitative study in which we conducted semi-structured interviews with multiple stakeholders, including program recipients, their families and officials involved in program implementation. We also sought relevant documents describing interventions in the two communities. The study was conducted following the Consolidated Criteria for Reporting Qualitative Research (COREQ).^15^

### Setting

In this project, we worked in two communities: community A, a formal settlement, and community B, an informal settlement.

Community A was set up for people affected by leprosy. The village is home to about 780 people, the majority of whom are people affected by leprosy and their families. The first settlers in the village were itinerant, homeless people in the state. The government resettled them in a colony and provided them with support. Over time, the colony grew in number and the existing support from the government was no longer sufficient, resulting in a decline in the state of health services and infrastructure. The settlement consists of apartments built by the state and non-governmental organisations (NGOs). The settlement includes a primary school and a health clinic.

Community B has been in existence for >6 decades. It exists around a specialised leprosy hospital that was sited within the community. The hospital serves as a referral point for leprosy patients from many states across the country who require advanced treatment. Most of the inhabitants were patients who came for leprosy treatment at the hospital and decided to continue living there instead of going back to their former homes. The community has a primary school and a borehole. This community has been neglected and the inhabitants live in fear of the possibility that the government could evict them at some point since they do not have title to the land.

### Intervention

We focus here on two programs, one implemented at each site, communities A and B. Both programs were implemented by the same NGO.

Both projects began in 2013 and ended in 2019. The project proposals and final reports confirm that SHGs existed in each community before the interventions were initiated. These SHGs pursued health and livelihood activities. The project reports indicated that, among other interventions, at least 10 SHGs were assisted in securing improved seedlings, fertilisers and business loans totalling about ₦150 000 (approximately US$100) per person through linkage to the loan programs.

Both projects were implemented to foster inclusive development by addressing the intersecting challenges of public health, social exclusion and economic marginalisation affecting vulnerable groups such as people affected by leprosy, persons with disabilities and women. Through a rights-based approach, the projects aimed to deliver interventions that enhanced community well-being, expanded access to economic opportunities and strengthened protections for marginalised populations.

In both cases, the major components of the interventions included seed capital for small businesses, support for farming and animal husbandry and skills acquisition training in areas such as tailoring, carpentry and painting. The interventions were targeted to specific groups. Delivery of the interventions was conducted in collaboration with NGOs, government entities and community members, among other stakeholders. This collaboration was made possible through the establishment of a Project Implementation and Advisory Committee. Community involvement was further encouraged through the formation of a Central Community Management Committee, which consisted of community leaders, women leaders and youth leaders. The participatory methods were designed to promote community participation and ownership of project outcomes, with the expectation that this ownership would significantly contribute to the sustainability of the projects even after funding concluded.

### Data collection

This was part of a broader study involving sites from India, Nepal and Nigeria. The protocol for the study has been published elsewhere.^13^

#### Documents retrieval

Between July 2020 and April 2022, we contacted program managers who were involved in projects between 2013 and 2019, as well as leaders of the SHGs, and requested relevant documents that described the interventions and how they were delivered. We also searched for the described projects on the repository database of the funding organisation at www.glasscubes.com. These documents informed the above intervention description.

#### Semi-structured interviews

Study participants were identified by a purposive sampling technique. Community participants were identified through the community gatekeepers (village heads) and were invited to participate in the study. The gatekeepers were tasked with nominating eligible participants on the basis that they were involved in the intervention and were able and willing to share their experiences. The research program managers identified individuals who were involved in implementation of the interventions. Interviews were conducted to elicit their description of the programs and to provide any evidence on the effects and sustainability thereof. Informed consent forms were signed by participants after they had been thoroughly briefed about the study and provided with participant information sheets, which were translated into Hausa. All their questions were addressed and they were given up to 24 h to decide whether to participate in the study.

We developed semi-structured interview guides (Supplementary Appendix 1) with prompts and probes directed at each of the two participant groups. The interviews covered topics such as community information, details about the SHG, participants’ roles and experiences in the intervention and their knowledge of the sustainability of the interventions, with relevant examples. The interview guides were translated into Hausa and subsequently back-translated into English to ensure accuracy.

To mitigate social desirability bias, strategies suggested by Bispo^16^ were followed: data triangulation was applied during participant selection, categorising them as insiders or outsiders; interview questions were carefully formulated to enable participants to comfortably share their perspectives; interviews were conducted in an atmosphere that ensured participants’ privacy; confidentiality was maintained by concealing participants’ identities; interviews were scheduled in advance, with participants informed to avoid unplanned sessions; the interviewer received adequate training and was familiar with the participants’ language and culture; probes and prompts were used to minimise participants’ biased opinions or views; and researchers adhered to ethical, political and social standards, adopting a neutral attitude.

PT conducted all interviews in either Hausa or English, depending on the respondent’s preference. PT was employed by the implementing organisation but was not involved in delivering the interventions. Prior to conducting the interviews, PT completed postgraduate training in qualitative and comparative research methods in health at the University of Warwick.

RJL conducted interviews with two external participants: the head of the implementing organisation and a representative of the funding organisation. The interviews lasted from 20 min to 2 h.

A digital recording device (ICD-PX470, Sony, Tokyo, Japan) was used to record the interviews, which were later transcribed and translated into English. PT also recorded observations as field notes.

### Analysis of interviews

We used the NVivo qualitative research software (Lumivero, Denver, CO, USA) to analyse all the data generated from the study using the reflexive thematic analysis method.^17^ OI, PT and SC open-coded five rich transcripts to generate initial codes. The independently developed codes were discussed and a code book developed. OI analysed all interview transcripts and discussed findings and questions with the research team, including PT and FG.

Findings were synthesised and compared across and within groups. We looked for evidence on the extent to which the initiatives were sustained after the funding was withdrawn. In establishing sustained activities, we focused on activities or inputs that participants themselves could clearly link to the interventions being studied. We specifically looked to identify similarities and differences between the groups in terms of methods or philosophies and reviewed factors that might promote sustainability or act as barriers to sustainability. We also identified the contextual factors that participants considered to have influenced sustainability and why and how what happened in the groups influenced sustainability.

## Results

### Document analysis

We retrieved documents (n=18) that described or evaluated the self-help interventions. The documents were obtained from the program’s records of the implementing organisation in central Nigeria. The obtained records included the approved project proposal that was submitted to the funders for each of the two communities and the proposals for extension of the program after the first phase of the funding elapsed (n=2). We also obtained the implementation report documents that were submitted to the funders by the implementing organisation for the two phases of the implementation, both at mid-term and at the conclusion of the intervention (n=8). Other documents obtained were the final evaluation reports (n=4) and documents of registration of the SHGs with the government of Nigeria (n=2).

### Interviews

We also conducted qualitative interviews (n=40); 32 with community members tagged as ‘insiders’ (n=16 in each community) and 8 with ‘outsiders’ who were involved in the administration or funding of the interventions. An overview of participants is presented in Table 1.

**Table 1.** Participants interviewed.

Category of interviewed	Number interviewed (N=40)		
participants	Male	Female	Total
Community A			
Insiders			
SHG members	5	5	10
Families of SHG members	2	3	5
Community leader (Mai’angwa)	1	0	1
Outsiders			
Government officials	1	1	2
Health officials	1	0	1
Community B			
Insiders			
SHG members	5	5	10
Families of SHG members	3	2	5
Community leader (Mai’angwa)	1	0	1
Outsiders			
Government officials	1	0	1
Health officials	0	1	1
Intervening NGO officials	2	0	2
Funding organisation official	1	0	1

Through interviews and document reviews, we sought to understand the community contexts, their experiences of interventions and identify evidence of sustainability and contributing factors.

### Interview findings

#### How their lives were impacted by leprosy

Members of each community seemed to experience a leprosy diagnosis in distinctly different ways. For people in community A, while leprosy disrupted their lives, including through amputations and restrictions in activities and socialisation with the public, there were no mentions of relocation or separation from family because of leprosy. However, for people in community B, the most discussed effect of leprosy in their lives was the need to leave home.

Honestly, this sickness has affected my life so much because you are just there with your family and friends and then suddenly you came down with this illness, and you now have to leave home and settle down here.—Participant B6

#### The living situation in the two communities

In both settlements, participants reported discontent with their living situation. Members of community A expressed continuing needs in their community, these included needing more land for agriculture, greater government support, education and health needs, provision of food and financial support. The most mentioned of these was the need for educational support for their children. From the respondents, we learned that children from the community had access to some education, often primary education, but struggled with further education.

We need help, you see, we have challenge with the education of our children. After their primary education, the fees keep on increasing and we are not able to afford that. We need help in funding the education of our children. There are even those that reached the final of their secondary school but were unable to finish because there is no money. — Participant A4

However, in interviews with members of community B, all but two respondents felt that their most pressing need was for permanent accommodation. Even though some participants talked about three generations of their family living on the land, they faced constant threats of eviction. A few participants referred to the arrangements made for community A as desirable for them. However, it is unclear how they gained an awareness of the existence of community A as a government supported community.

I want to talk about our major problem in this community. We are constantly reminded that this land that we settled in is not ours and that we will soon be asked to leave but we have nowhere else to go. This is the place of our birth. For almost all of us, our parents have been rejected from where they came from, and they settled here and gave birth to us here. If we are asked to leave now, where do we have to go to? — Participant B2

#### Community relationships

It is well established that people affected by leprosy often face stigma and discrimination in their communities and homes. Participants in both communities shared experiences of discrimination from their families prior to treatment and from the surrounding non-leprosy-affected communities. There was a noted difference in participants’ reporting of their current situation. Community A participants were more likely to report continued discrimination than community B participants. In community B, most participants reported that discrimination had decreased and they had improved relationships with the broader community. Participants did not always have any rationale for these changes, but a few linked the development to increased knowledge about their condition in the community and improved hygiene and ulcer care after participating in leprosy mission education sessions.

The relationship has been cordial because the people around understands since they are people that are mostly affected. And when we go outside here, the stigmatisation has reduced, and people have understanding now that things are no longer as they were. We have been trained to look after ourselves and have good hygiene, so, people can accept to live with us. In past people taught that they will contract the disease if they don’t avoid us, and that is not even stigmatisation because it is fear. Nowadays we have been treated again and again and people have the awareness that whenever you see a person with leprosy that has been treated, he cannot infect another person, that is why now we have the liberty to associate freely with other people. — Participant B1

#### Sustainability of empowerment programs

In community A, participants spoke about training offered to members of the SHG and seed funds to start small businesses. The number of SHGs formed was inconsistently reported, ranging from 3 to 16. However, from most of the interviews, it was evident that groups were based on gender and age: men, women and youth. From the responses, it was also clear that men’s groups were considered less successful than the other two groups. The most consistently reported activity was the provision of sewing machines and tailoring training provided to at least five women. Multiple participants reported that the machines were still in working order and being used to participants’ (financial) benefit at the time of the interview. We consider the inclusion of beneficial utilisation of sewing machines as an indication of sustained activity. Some participants also explicitly mentioned improved financial situations because of using their sewing machines. However, two participants spoke about sewing machines being sold off or no longer working.

…not all of them are using the resources judiciously. There are some that even sold off their sewing machines instead of using them for the tailoring business that they were taught. — Participant A11, family member of a beneficiary

 

…brought sewing machines, five of them…and paid money for the training of five women on sewing…I commend our women as well because none of them have sold off any of the machines, because they are still available and very useful for about 5 years now. They are using them and are benefitting from them still. — Participant A2

The participants in community A also spoke frequently about the provision of grinding machines for grains. As with the sewing machines, most participants stated that the grinding machines were still in working order. A range of other economic activities were discussed, including trading clothing or foodstuffs, rearing livestock, cake baking and carpentry. However, only a few participants recalled receiving material support or training to facilitate these activities. Moreover, given the long history of interventions in the community, it is unclear when these activities were implemented, whether as part of the above or an earlier initiative. Only the sewing and grinding machine provision was clearly dated by participants as occurring within the 5-y time frame of the index intervention.

Participants from community B also recounted similar training and empowerment programs with two distinct differences. First, participants spoke about SHG/NGO intervention facilitating the provision of essential infrastructure for the community, namely a school, a borehole for clean water and electricity. From the interviews, it was not made clear how the NGO facilitated the provision of electricity. There was no mention of alternatives to the national grid, such as solar panels or generators. The second difference was the discussion of a scheme to provide at least five tricycles in the community. While all participants spoke positively of the scheme, by all accounts the tricycles had all been sold after about 2 y, as the community lacked the skills to maintain them.

The business was good and we have been sustained by it, but they began to get old and needed to be repaired very often. It was no longer sustainable to keep on spending to repair them. That was how they were sold and the money shared among us…More were purchased, but later, we could not continue to sustain the business. — Participant B8

Regardless of the outcomes, participants in both communities expressed a strong preference for skills training over the provision of handouts like food. In community B, participants commonly referred to the common adage of ‘teaching a man to fish’. Despite this belief, most participants at some point in their interviews indicated the need for continuous support for the communities, including the provision of foodstuffs. This is perhaps linked to an often-expressed idea that regardless of how equitably training and business support opportunities are distributed, there will always be people who do not benefit from the program.

The truth is that even if it rains money, not everyone will get but some will get. So, you see, when I complained that I did not benefit, I still feel that it wasn’t my luck. It also gives me joy to see someone close to me benefit from something good because I know that someday it will be my turn.—Participant B11, family member of a beneficiary

#### Locus of control

One striking finding was related to participants’ sense of control over their life situation and outcomes. The interventions sought not only to improve the socio-economic standing of participants but also to empower them. Although some respondents spoke about being better able to advocate for themselves, most participants explicitly discussed the need for advocates and support. However, in this context there was a clear dependence on a divine power. Participants frequently thanked God for the support they had received and the successes they had achieved. Included in this were prayers of blessing for the organisations that supported them. Participants also commonly mentioned praying about future opportunities and success for themselves and their families.

Very well. Everything about the group is okay for us. Our prayer is for God to keep us progressing. We are praying for more support so that our children will do well, and our elders will be able to rest. — Participant B9

 

Praise be to God. The intervention was exactly what we wanted. All that we have to say is, may God bless those that assisted us, and for those that are capable but not willing, we pray that God will give them the will to support us.—Participant A8

Discussions with the funding organisation were unsatisfactory in the sense that the interviewee was not involved in the original projects and did not have a clear answer regarding what the funder had planned with respect to sustainability.

## Discussion

Our study aimed to determine the sustainability of economic interventions within two unique communities in central Nigeria. We found strong evidence that some programs had been implemented in the two communities, but program specifics were vague. However, evidence of sustainability was mixed, with participants unanimously agreeing that many activities had not been sustained. Community A certainly sustained sewing activities, but it was hard to identify sustainability in community B.

Reflecting on what economic interventions were delivered, it was clear that participants are beneficiaries of multiple programs and recollecting the details about interventions was a difficult task. However, we were able to establish that there were three specific income-generating activities that participants associated with the implementing NGO. These were the provision of sewing machines and training in their use, grinding machines and tricycles. These observations were consistent with data extracted from the project documents from the referenced time. It was clear, however, that sustainability was impeded by a lack of maintenance and access to repairs. The most provided reasons for interventions being discontinued were the inability of recipients to maintain the working order of the equipment. For other mentioned interventions, such as cattle rearing, participants seemed to face challenges that necessitated sale of the animals. This seems to be consistent with evidence on the outcomes of such empowerment interventions, even during the funding period.^14,18^ However, the long-term failures of these activities had not been previously reported. Most studies on SHGs describe the outcomes and the end of the funding period and do not examine the extent to which the activities and outputs are sustained into the future.^19,20^ Likewise, studies examining the literature have found few follow-up studies examining sustainability. Specifically in leprosy, Shrestha et al.^12^ found improved ulcer management, enhanced well-being, social integration and economic empowerment among marginalised populations in a leprosy community in Nepal. Gosh et al.^14^ identified four primary factors influencing the sustainability of SHGs in disaster-affected communities in India: managerial functions, trust, funds utilisation and financing. Lastly, Choudhury et al.^13^ conducted a review covering SHGs, which found only one study by Sondaal et al.^21^ that followed up a community self-help intervention in Nepal.

A key component of these empowerment SHGs is developing participants’ ability to become self-sufficient. However, in our study we found that although participants discussed the importance of self-sufficiency, they also almost unanimously discussed needing continuous support from organisations and individuals. They also had an externalised locus of control and discussed feelings of fear about their future. This is consistent with findings from a review by Somar et al.^22^ that found the mental well-being of persons affected by leprosy and their families is often poor, including feelings of fear and low quality of life. In a related finding, the context of our two communities contributed to participants’ continued needs. In community A, the primary challenges seemed to be limited economic mobility and access to education, which had implications for the next generation, while in community B, the pervasive fear of eviction and lack of land tenure created an environment of profound instability for individuals and families. Although we found no direct evidence to suggest that these environments influenced the sustainability of SHG activities, they were significant to our participants and influenced their overall quality of life.

This presents a conundrum: is it possible for marginalised individuals with limited education and low socio-economic status to achieve self-sufficiency in a context like Nigeria? Nigeria is a complex country that experiences what is known as the resource curse or the paradox of plenty.^23^ Although the country is resource-rich, citizens fail to benefit from the wealth, as public infrastructure and systems are weak.^24^ All participants shared concerns that children in their communities faced difficulties accessing education. In a context where even educated individuals face limited opportunities for social and economic mobility, it is difficult to imagine how leprosy-affected individuals can accomplish self-sufficiency. Perhaps the question to be asked is not about short-term empowerment but about developing infrastructure and systems that could potentially support self-sufficiency in the long term.

### Strengths and limitations

One notable strength of this study is its use of triangulation, which allowed us to gather comprehensive insights into the sustainability or lack thereof of the interventions. This was achieved through interviews with various categories of respondents, including direct beneficiaries, their family members, implementing program managers and donors. Furthermore, these findings were largely corroborated by the observations and assessments of field researchers.

The study also has some significant limitations that should be taken into consideration. A key limitation is related to the challenges of conducting retrospective research among our population. Participants often are unable to write or keep records of activities that have potentially influenced their ability to accurately reflect on events that occurred up to 5 y ago. Our participants are also relatively unfamiliar with qualitative research, and most struggled with responding to questions in extensive detail. We also had an additional challenge of multiple similar co-occurring interventions, which might have presented challenges for participant recall. The research was conducted by the staff of the implementing NGO, and this may have contributed to the risk of social desirability bias. However, working with the NGO was crucial to ensuring engagement with the community, and an external researcher would have had difficulties identifying and connecting with the relevant stakeholders.

## Conclusions

Our study sought to ascertain the sustainability of economic interventions within two unique communities in central Nigeria. We found that there was strong evidence of the sustainability of one intervention at one site. However, there is little to show for the interventions that seem to have produced little return on investment. We propose that the sustainability of economic interventions of SHGs can only be achieved when disenfranchised communities have access to fundamental infrastructure, such as schools for their children. Donors should pay much more attention to the issue of sustainability and build plans for sustainability into their funding procedures.

## Supplementary Material

ihaf140_Supplemental_File

## Data Availability

All the data obtained and/or analysed during the current study are available from the corresponding author upon reasonable request.
