# Supplementary material for: How sustainable are self-help interventions in the post-funding period? Qualitative findings from integrated leprosy communities in Nigeria
Source: Int Health. 2025 Dec 19;18(5):719–25. doi: 10.1093/inthealth/ihaf140 (PMC13530288; doi:10.1093/inthealth/ihaf140)
Supplement: Supplementary file 1 [file ihaf140_supplemental_file.docx]

**Appendix 1: Interview Topic Guides**

*Jimlar na 1: Jagoran Batutuwan Hira*

•	We are interested in finding out about [name of the programme]. Could you tell us about your experiences of that programme/community?

*Muna niman bayani a kan [sunan shiri]. Menene kuka sani akan wanan shiri/unguwa?*

a.	What was/is your role in the process/community?

*Wanda irin rawa ne kuka taka ko kuma menene matsayin ka/ki akan wanan shirin/unguwa?*

b.	What was/is the aim of the programme/community? Prompts: self-help/self-care.

*Menene manufar wanan shirin/unguwa? Ra’ayoyi: taimakon kai/kula da kai.*

c.	How was/is the [name of the programme] funded and administered? Prompts: political oversight, liaison, management - facilitators/group leaders, chair, meeting frequency, gender make up, attendance issues.

*Ta yaya aka gudanar da wanan shirin? Ra’ayoyi: bin dindigin siyasa, hadin kai, ta hanun masu gudanarwa/shuagabanin kungiyoyi, mitan ganawa.*

d.	Who was/is the programme/community designed for? Prompts: people with/without leprosy, disability, marginalised people.

*Don wa aka sara wanan shirin/unguwa? Ra’ayoyi: Kutare/marasa kuturta, nakasasu, mutanin da aka watsi da su.*

e.	How were/are people selected for the programme/community?

*Ta yaya aka zaba mutane zuwa wanan shirin/unguwa?*

f.	What components were/are included in the programme/community? Prompts: self-help, savings, bank accounts, training, employment, water, sanitation, hygiene work.

*Wanda kasusuwa ne suke a cikin wanan shirin/unguwa? Ra’ayoyi: taimakon kai, tanadi, asusun banki, horo, samun aiki, ruwa, tsafta, aikin tsabtacewa.*

•	What were your/participants’ experiences during and after the programme?

*Wanda irin ganewa ne ka/kin samu ta hanya wanan shirin?*

•	What are your experiences of living in the community (question for Alheri group)

*Yaya zaman ka/ki a nan ya kasance (tambaya zuwa gay yan gidan Alheri).*

a.	Did you/people face any issues in being part of the self-help groups? If so, what were they? Prompt: stigma, distribution of self-care materials, envy/resentment from people not included.

*Wanda masaloli ne kuka fuskanta a kungiyar taimakon juna? Ra’ayoyi: kiyaya, rabawar kayan taimakon kai, kishi daga wayanda ba’ayi tare da su ba.*

b.	What were/are the successes of the groups? Prompts: reduction in ulcers, micro-enterprise, community development.

*Wanda irin nasarori ne aka cinma a kungiyoyin ku? Ra’ayoyi: roguwar ciwon ulcer, kananan masana’antu, cin gaban al’umma.*

c.	Has the group and/or group activities been sustained since the funding ceased?

*Tun da aka tsiyar da shigowar kudade domin wayenan aikatun, kun iya cin gaba da ayukanku ko kuma a’a?*

d.	What would improve (the longevity of) the self-help groups?

*A ra’ayin ka/ki, me zai sa kungiyar taimakon juna ta ci gaba?*

•	Are you aware of any programme evaluations/sustainability studies that have been done?

*Ka/ki na da sani ko akwai wani shiri na musaman da aka gudanar domin bincike yanda za’a samu cin gaban wanan shirin?*
